# Thiamine Diphosphate Supplementation as a Heat-Stress Mitigation Strategy for Hair Male and Female Lambs in Feedlot: Physiological Responses, Growth Performance, and Carcass Traits

**DOI:** 10.3390/ani15213143

**Published:** 2025-10-29

**Authors:** Ulises Macías-Cruz, German Castillo Cristóbal, Leonel Avendaño-Reyes, María de los Ángeles López-Baca, José A. Roque-Jiménez, Miguel Mellado, César A. Meza-Herrera, Ricardo Vicente-Pérez, Marisol López-Romero, Nallely Rivero-Pérez

**Affiliations:** 1 Instituto de Ciencias Agrícolas, Universidad Autónoma de Baja California, Valle de Mexicali 21705, Baja California, Mexico; umacias@uabc.edu.mx (U.M.-C.); germa.castillo@uabc.edu.mx (G.C.C.); lar62@uabc.edu.mx (L.A.-R.); jose.roque@uabc.edu.mx (J.A.R.-J.); mlopezromero5@gmail.com (M.L.-R.); 2 Instituto de Investigación en Ciencias Veterinarias, Universidad Autónoma de Baja California, Mexicali 21386, Baja California, Mexico; 3 Departamento de Nutrición Animal, Universidad Autónoma Agraria Antonio Narro, Saltillo 25315, Coahuila, Mexico; mmellbosq@yahoo.com; 4 Unidad Regional Universitaria de Zonas Áridas, Universidad Autónoma Chapingo, Bermejillo 35230, Durango, Mexico; cmeza2020@hotmail.com; 5 Centro Universitario de La Costa Sur, Universidad de Guadalajara, Autlán de Navarro 48900, Jalisco, Mexico; ricardo.vperez@academicos.udg.mx; 6 Instituto de Ciencias Agropecuarias, Universidad Autónoma del Estado de Hidalgo, Tulancingo de Bravo 43660, Hidalgo, Mexico; nallely_rivero@uaeh.edu.mx

**Keywords:** dietary energy efficiency, hair breed sheep, meat quality, sexual dimorphism, thiamine diphosphate, thermography

## Abstract

**Simple Summary:**

High summer temperatures in desert regions decrease weight gain, feed efficiency, carcass weight, and meat tenderness in feedlot sheep, which is associated with insufficient availability of dietary energy by increasing energy requirements for thermoregulation. So, hair male and female lambs were fed thiamine diphosphate to evaluate its effect as glucogenic cofactor on physiological variables, feedlot growth, carcass traits and meat quality under hot desert conditions. Thiamine improved heat loss along the body surface, without changing rectal temperature and respiratory rate. Furthermore, it reduced feed intake in females but not in males. Regardless of gender, thiamine did not modify growth rate, carcass yield, fat deposition, and color and hardness in meat. Therefore, this heat stress mitigation strategy effectively increased dietary energy efficiency in ewe lambs but was ineffective for male lambs.

**Abstract:**

Twenty Dorper × Katahdin lambs (10 males and 10 females) were distributed in a 2 × 2 factorial arrangement under a randomized complete block design to evaluate the effects of thiamine diphosphate (TD) supplementation (0 vs. 250 mg/kg feed) and gender (males vs. females) on physiological responses, feedlot performance, carcass characteristics, and meat quality in a hot desert environment. The average temperature and temperature–humidity index recorded during the study were 33.60 °C and 35.89 units, respectively, indicating an extremely severe heat stress environment for lambs. Study variables were not affected (*p* ≥ 0.12) by the TD × gender interaction, except for dry matter intake (DMI; *p* = 0.02) and some head temperatures (*p* ≤ 0.05) and carcass zoometric measurements (*p* ≤ 0.05). In females, but not in males, TD decreased DMI and increased thorax depth, as well as eye, ear, and forehead temperatures. Overall, TD increased (*p* ≤ 0.05) surface temperatures of neck, shoulder, loin, rump, forelimb, testicles, vulva, anus, and perineum without affecting (*p* ≥ 0.58) rectal temperature and respiratory rate. Supplemental TD did not affect (*p* ≥ 0.16) growth rate, feed efficiency, carcass weight and yield, *Longissimus thoracic* muscle area, backfat thickness, internal fat deposition, wholesale cut yields, and meat quality traits. In conclusion, in hair ewe lambs but not in male lambs, TD supplementation at a dose of 250 mg/kg of feed in the fattening diet is an HS mitigation strategy that improves dietary energy efficiency for growth and carcass mass deposition. Furthermore, thiamine increases heat losses through the body surface, regardless of gender.

## 1. Introduction

Hair sheep are characterized by growing and producing meat under summer heat stress (HS) conditions in arid and desert regions [1]. Despite this behavior, the summer HS decreases the growth potential of fattening male and ewe lambs of these breeds by 20 to 30%, which negatively impacts muscle deposition, carcass weight, and meat quality [2,3]. These adverse effects of HS on growth and carcass traits are not ascribed to decreased feed intake, as in other sheep breeds, but rather to a redistribution of dietary energy that favors thermoregulation processes more than growth [4]. Given that these lambs have low availability of metabolizable energy (ME < 1.8 Mcal/kg dry matter) for growth, and their feed intake is maintained under HS, an alternative strategy for mitigating HS could be dietary supplementation of glucogenic cofactors.

Thiamine diphosphate (TD) is a glucogenic cofactor derived from vitamin B1, which functions as a coenzyme for enzymes involved in the cell’s bioenergetic processes that lead to adenosine triphosphate (ATP) synthesis [5]. Thus, the enzymes pyruvate dehydrogenase, α-ketoglutarate dehydrogenase, transketolase, and branched-chain α-ketoacid dehydrogenase require TD as a cofactor in glycolysis and oxidative carbohydrate decarboxylation [6,7]. This response, in turn, stimulates greater ATP production and metabolizable energy by promoting better glucose utilization, as well as the availability of gluconeogenic substrates [8,9]. Based on the above, this glycogenic cofactor can enhance dietary energy efficiency mainly by aiding cells in converting ingested carbohydrates into energy through metabolic pathways (glycolysis) that optimize ATP production from glucose oxidation; likewise, if necessary, it also contributes to promoting the synthesis of gluconeogenesis precursors from fatty acids and short-chain amino acids from the diet [6,10]. In addition, it counteracts inflammatory processes and oxidative stress [11], two common problems triggered in sheep exposed to high environmental temperatures (Ta) [12]. In this way, TD in heat-stressed lambs has the potential to enhance not only dietary energy efficiency but also redox status, immune system function, and overall welfare.

To date, some studies have evaluated supplemental TD on the productive performance and carcass traits of fattening lambs, but only under thermoneutral conditions (12 to 30 °C), reporting inconsistent findings. Two experiments were conducted with Rambouillet wethers and female lambs using the same thiamine treatments (0, 50, 100, and 150 mg/head), and the difference between them was group or individual feeding [13]. Supplemental thiamine in group feeding, but not in individual feeding, was effective to increase weight gain, feed efficiency, dry matter intake (DMI), and carcass weight in a dose-dependent manner, while other carcass traits were unaffected (i.e., conformation, *Longissimus thoracis* [LT] muscle area, backfat thickness, and quality grade). In Afec-Assaf male lambs, subcutaneous injections of high-dose thiamine improved glucose metabolism and hepatic glycogen storage with no growth rate improvement [10]. Note that while TD feeding has not been investigated in heat-stressed fattening lambs of any breed, this vitamin has been shown to partially reverse the adverse effects of HS on milk production and quality, but not on DMI and body condition of lactating ewes [11]. This response advocates that TD can increase dietary energy efficiency and redirect this energy to improve productive variables of animals experiencing HS.

It is important to note that metabolic sexual dimorphism in fattening lambs could play an essential role in the effectiveness of TD to improve the availability of dietary energy for growth. The gonadal endocrine activity adjusts the energy and protein metabolism to prioritize the distribution of nutrients in different tissues according to gender [14]. While ovarian-synthesized estrogens favor the transformation of nutrients into adipose tissue rather than muscle tissue in ewe lambs, testicular testosterone in male lambs acts as a natural anabolic, prioritizing energy use for protein synthesis and lean muscle tissue deposition [15]. Thus, male lambs compared to female lambs under a feedlot systema show superiority in muscularity, growth performance, and carcass weight and size, but regularly lower carcass yield and body fat deposition, with contradictory gender effects on meat quality even though male lambs tend to accumulate more muscle glycogen [15,16]. Although HS partially or totally disrupts reproductive events in mammals by inactivating the endocrine reproductive axis with the activation of the hypothalamic–pituitary–adrenal axis [17], hair sheep appear to have reproductive resilience to HS, as both males and females can reproduce under high Ta of desert regions [18,19]. In this context, it was hypothesized that feeding TD to feedlot hair sheep improves productive performance, carcass traits, and meat quality without compromising physiological thermoregulatory capacity, especially noticeable in male lambs compared to females, under high summer Ta. Therefore, this study aimed to evaluate the effects of supplemental TD and gender on the physiological responses, feedlot growth, carcass traits, and meat quality of heat-stressed hair breed lambs during the summer in a desert region.

## 2. Materials and Methods

### 2.1. Study Site and Experimental Period

The study was conducted during the hottest summer months (July and August) at the Sheep Experimental Unit of the Institute of Agricultural Sciences, Autonomous University of Baja California (UABC), located in the Mexicali Valley, Baja California, Mexico (32.38° N and 115.28° W). The region presents a hot desert climate (BWh), with maximum temperatures exceeding 45 °C in the summer and minimum temperatures of 0 °C in winter; the average annual rainfall is 77.8 mm, mostly occurring in winter [20].

### 2.2. Animals and Pre-Experimental Handling

Twenty Dorper × Kathadin crossbred lambs (10 females and 10 males) were used and managed according to the Mexican Official Standards: NOM-051-ZOO-1995 [21] (establishes the humane treatment of animals during their mobilization), NOM-062-ZOO-1999 [22] (describes the management of production, care, use and health protection of laboratory animals), and NOM-033-SAG/ZOO-2014 [23] (specifies the methods for the humane slaughter of domestic and wild animals). Both protocol and field phase were also revised and authorized by the UABC Ethics Committee (Letter number: 047/2024-1).

All lambs received a single prophylactic handling, 30 d before starting the feedlot trial, as well as they were adapted to individual pens and to a basal experimental diet for 15 d. Prophylactic handling consisted of treating each animal with 3.0 mL of oral dewormer (Adbendal 10% Co^®^, Adler Pharma Laboratory, Jalisco, Mexico), 0.5 mL of A-D-E vitamins intramuscularly (Vigantol ADE Fuerte^®^, Elanco Animal Health Laboratory, Guadalajara, Jalisco, Mexico), and 2.5 mL of clostridium vaccine subcutaneously (Bovimune^®^ Clostri 10, LaPisa Laboratory, La Piedad, Michoacan, Mexico). The individual pens were built in an area of 2.0 m^2^, with cyclonic mesh walls and galvanized sheet shade installed at a height of 2.5 m on a dirt floor. Each pen was provided with a feed bunk and water trough. The experimental diet was formulated to meet the nutritional requirements for fattening lambs [24] by mixing the ingredients indicated in Table 1.

### 2.3. Experimental Design and Handling

**Figure 1:**
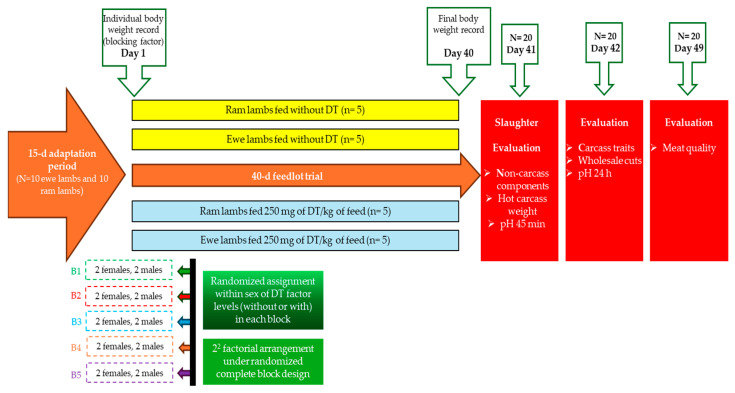
Diagram of the experimental design and evaluation of the study variables.

On the first day of the 40-d feedlot test, lambs were about 4 months old and were weighed individually (BW = 31 ± 2.8 kg). Blocks of two male lambs and two ewe lambs of similar BW were formed and then lambs of each block were randomly assigned within each gender to one of two dietary strategies: basal diet without or with 250 mg of TD/kg of feed. Overall, lambs were distributed in four treatment combinations (*n* = 5) according to a 2 × 2 factorial arrangement under a randomized complete block design (Figure 1): (1) males without TD, (2) males with TD, (3) females without TD, and (4) females with TD. It is worth mentioning that TD was mixed directly into the diet and was offered during the 40-day feeding trial. Feed was offered ad libitum daily, with a 15% feed refusal, and was served at 06:00, 12:00, and 18:00 h. Drinking troughs were refilled simultaneously with fresh, clean water, while the sheep’s health status was visually monitored in the morning and afternoon, with no sick animals detected. Once the feeding trial was completed, lambs were fasted for 12 h and then slaughtered by exsanguination at a slaughterhouse located at the study site.

### 2.4. Climatic and Physiological Variables

Relative humidity (RH), Ta and temperature–humidity index (THI) were recorded as climatic variables. The first two were measured with a thermohydrometer (Termotraker^®^, Culiacan, Sinaloa, Mexico) that was placed in the center of the experimental area at the height of the animals’ heads. This device was programmed to automatically record both Ta and RH every 20 min during the 40-d feedlot test. Upon completion, the data were downloaded to Excel^®^ to calculate the THI with the following formula [26]: THI = Ta − {(0.31 − [0.31 × RH]) (Ta − 14.4)}. For all climatic variables, average values per day and hour were calculated, and average daily maximum and minimum values were obtained.

**Figure 2:**
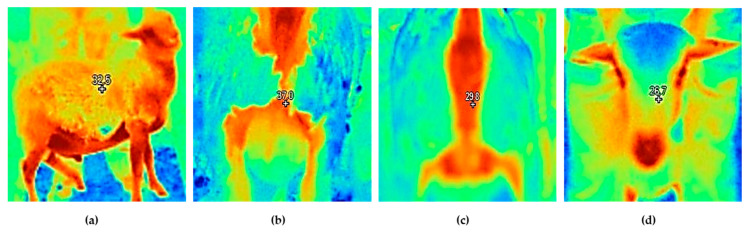
Thermographic images taken to assess body surface temperatures in lambs: (**a**) side view, (**b**) caudal view in male lambs, (**c**) caudal view in ewe lambs, and (**d**) front view.

Respiratory rate (RR), rectal temperature (RT), and surface thermography in different body regions were assessed at 06:00, 12:00, and 18:00 h on days 1, 10, 20, 30, and 40 of the feedlot trial. The number of intercostal movements per minute was quantified to determine RR, while a digital thermometer (Delta Track, Pleasanton, CA, USA) was inserted into the rectum to record RT. Finally, three thermal images per lamb were taken (front, side, and caudal; Figure 2) with an infrared thermographic camera (Fluke Ti401, Everett, WA, USA). These were downloaded to the computer to record the surface temperatures with the Smart View^®^ software (version 4.4., Fluke, WA, USA) in the following body regions: eye, ear, muzzle, forehead, nostrils, neck, shoulder, loin, belly, rib, flank, rump, leg, fore limb, hind limb, vulva, anal, rectal, perineum and testicles.

### 2.5. Feedlot Performance

All lambs were weighed individually on days 1 and 40 of the feedlot trial to record initial and final BW. The amount of feed offered and refused in each pen was weighed daily in the morning to calculate DMI. In addition, total weight gain (TWG), daily weight gain (ADG = TWG/fattening days), and feed efficiency (ADG/feed intake) were calculated.

### 2.6. Carcass Traits, Non-Carcass Components, and Wholesale Cut

All lambs were weighed before the slaughter to record slaughter BW. Once killed, lambs were bled, skinned, and eviscerated to record the individual weights of blood, head, skin, feet, testicles, heart, lung, liver, kidneys, spleen, full and empty gastrointestinal tract (GT), internal fats (omental, mesenteric, and kidney-pelvic-heart [KPH]), and hot carcass (HCW). While KPH fat weight was expressed as a percentage of the HCW, the weights of the other non-carcass components were expressed as a percentage of the empty live weight (EBW = slaughter BW − [full GT − empty GT]). Hot carcass weight was also expressed as a percentage of the EBW to calculate the carcass yield.

Subsequently, the carcasses were placed in a cold room at 4 °C to record cold carcass weight (CCW) and zoometric measurements (i.e., carcass length, thorax depth, chest circumference, and leg length and circumference). Then, carcasses were ribbed between the 12th and 13th to measure *LT* muscle area with a dot square grid of 64 mm^2^, as well as backfat thickness with a vernier. Finally, the right half carcass was weighed and dissected to measure the wholesale cut yields as described by Avendaño-Reyes et al. [27]. Briefly, the individual weights of neck, shoulder, loin, plain loin, rib, breast, and flank, leg, forequarter, and hindquarter were expressed as a percentage of the half carcass weight to calculate their yields.

### 2.7. Meat Quality

The carcass pH was recorded at 45 min and 24 h *postmortem* by inserting a penetration electrode into the loin (12th and 13th rib), connected to a portable pH meter (HI-98140, Hanna Instruments, Woonsocket, RI, USA). Subsequently, the *LT* muscle was dissected and vacuum-packed for aging during 7 d at a temperature between 0 and 4 °C. This muscle was unpacked and subjected to blooming for 30 min before evaluating the meat quality parameters: color, pH, cooking loss, Warner-Bratzler shear force (WBSF), and water-holding capacity (WHC).

The color was measured in triplicate at different points on the surface of each muscle using a portable colorimeter (CR-400, Konica Minolta Sensing, Inc., Osaka, Japan; D65 illuminator and 10° observer). Lightness (L*), redness (a*), yellowness (b*), Chroma (C*), and hue angle (h*) were color variables recorded after calculating an average of each one per muscle. Subsequently, 5 g of muscle and 25 mL of distilled water were homogenized (liquefied) for one minute, and then a pH meter for liquids was introduced into the mixture to measure pH (HI-2210, Hanna Instruments, Woonsocket, RI, USA). The pH was determined in duplicate to calculate an average for each muscle. For cooking loss and shear force, one steak was weighed, cooked until a core temperature of 71 °C using an electric grill, and finally chilled to room temperature for 20 min. The cooled steak was weighed again, and this weight was subtracted from the steak’s weight before cooking it; the difference in weight was expressed as a percentage of the uncooked steak’s weight to record cooking loss. In addition, the cooled steak was cut to obtain three cubes measuring 1.27 cm per side. These cubes were shared longitudinally with respect to the muscle fibers using a Warner-Bratzler shear force device (Salter 235, Manhattan, KS, USA). Finally, the WHC was determined following a standard methodology [28], which consists of weighing 3 g of meat and suspending it on porous fabric within 50 mL tubes es to be centrifuged and weighed again post-centrifugation. Thus, the WHC of the meat was calculated by expressing its post-centrifugation weight as a percentage of its initial weight. Both WBSF and WHC were measured in triplicate, so averages per muscle of each of them were recorded.

### 2.8. Statistical Analysis

All data were subjected to analysis of variance using procedures from the SAS statistical package (SAS Institute Inc., Cary, NC, USA; version 9.4). Productive performance, carcass characteristics, non-carcass components, wholesale cut yields, and meat quality were analyzed with the GLM procedure, where the models included fixed effects of block, TD supplementation, gender, and the TD × gender interaction; additionally, animal was included in the models as a random effect. Physiological variables were analyzed with the MIXED procedure using the same model, but the hour of the day was included as a repeated measurement factor over time. In this model, different variance-covariance structures were tested to select the best fit based on the AIC and BIC values closest to zero. Means were compared with the PDIFF option of SAS, declaring differences at *p* ≤ 0.05.

## 3. Results

### 3.1. Environmental Conditions

**Figure 3:**
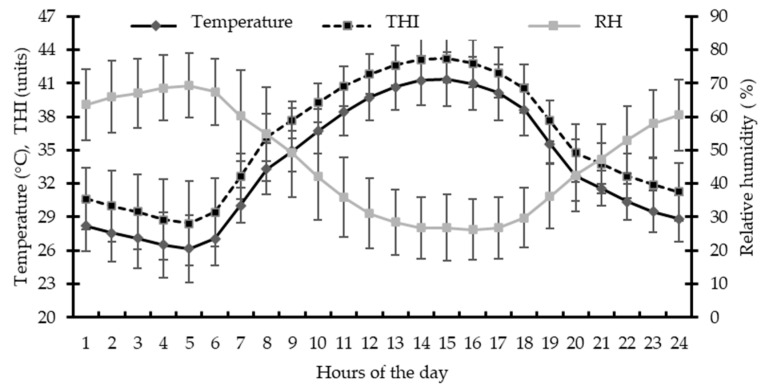
Circadian variation in environmental temperature, relative humidity (RH), and temperature–humidity index (THI) recorded during the feedlot trial.

The average Ta and RH recorded during the 40-d feeding period were 33.6 ± 1.4 °C (25.9–41.8 °C) and 47.41 ± 8.50% (23.8–72.3%), respectively, leading to an average THI of 35.89 ± 1.69 units (28.0–43.55 units). Daytime Ta remained above 30 °C and lasted until 22:00 h, while the THI did not drop to a thermal comfort level (<22.2 units) at any time of day (Figure 3).

### 3.2. Effects of Thiamine Diphosphate

**Figure 4:**
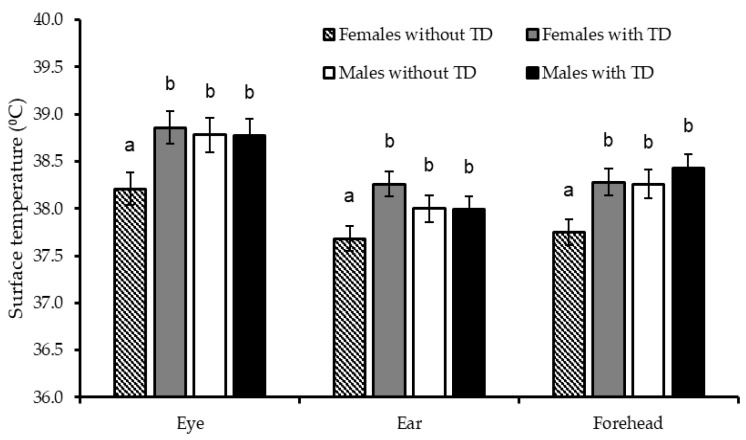
Effect of the thiamine diphosphate (TD) × gender interaction on surface temperatures in different regions of the head of Dorper × Katahdin lambs fattened under heat stress conditions. Differences (*p* ≤ 0.05) between the combinations of factor levels are indicated by different letters on the mean bars for each variable.

No interaction between TD, gender, and/or hour of the day (*p* > 0.05) was detected for RT and RR; likewise, these physiological variables were not affected (*p* ≥ 0.78) by the supplemental TD alone (Table 2 and Figure 4). Some body surface temperatures were only affected (*p* < 0.05) by the TD × gender interaction; particularly, TD in lambs, but not in male lambs, increased eye, ear, and forehead temperatures by more than 0.50 °C. Regardless of gender, TD increased (*p* ≤ 0.05) surface temperatures of neck (+0.45 °C), shoulder (+0.43 °C), loin (+0.38 °C), rump (+0.42 °C), forelimb (+0.44 °C), anal (+0.47 °C), vulva (+0.71 °C), perineum (+0.80 °C), and testes (+1.62 °C), as well as, tended to increase them in rib (*p* = 0.09; +0.37 °C), flank (*p* = 0.07; +0.41 °C) and hind limb (*p* = 0.09; +0.33 °C). Surface temperatures in the rest of the body regions were not affected (*p* ≥ 0.11) by TD feeding.

**Figure 5:**
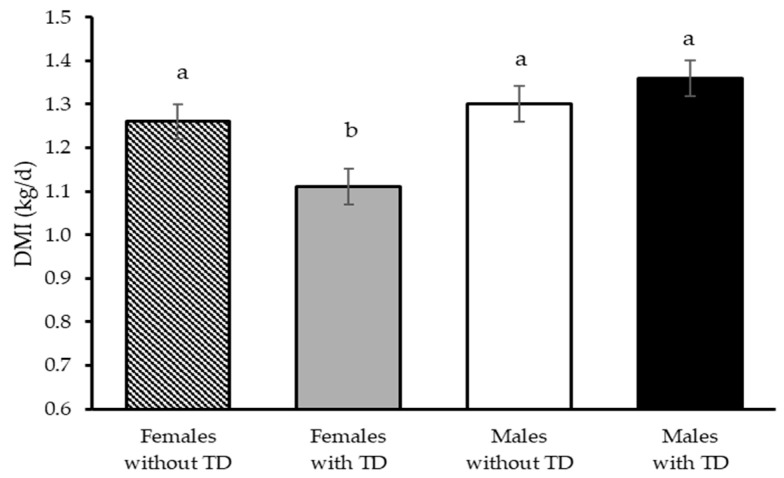
Effect of the thiamine diphosphate (TD) × gender interaction on the dry matter intake (DMI) of Dorper × Katahdin lambs fattened under heat stress conditions. Differences (*p* ≤ 0.05) between the combinations of factor levels are indicated by different letters on the mean bars.

In feedlot performance, the TD × gender interaction affected (*p* = 0.02) only DMI; TD decreased intake by 12% in ewe lambs with no effect in male lambs (Table 3 and Figure 5). Regardless of gender, there were no changes (*p* ≥ 0.28) in weight gain, feed efficiency, and final BW due to TD.

**Figure 6:**
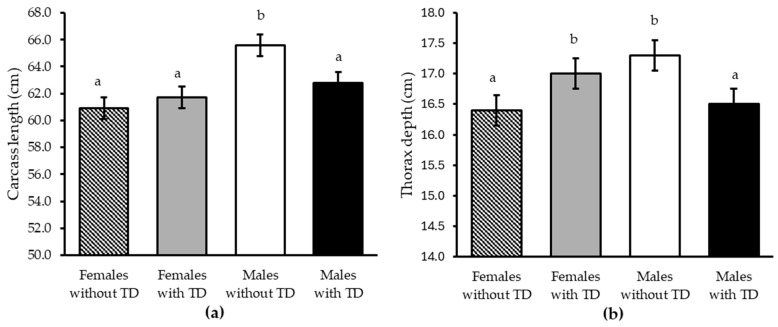
Effect of the thiamine diphosphate (TD) × gender interaction on the carcass length (**a**) and thorax depth (**b**) of Dorper × Katahdin lambs fattened under heat stress conditions. Differences (*p* ≤ 0.05) between the combinations of factor levels are indicated by different letters on the mean bars for each study variable.

In carcass characteristics, both TD alone or interacting with gender did not affect (*p* ≥ 0.15) carcass weight and dressing percentage, LT area, chest circumference, leg length and circumference, back fat thickness, and KPH and omental fat deposition (Table 4). Nevertheless, a 23% reduction (*p* = 0.03) in mesenteric fat accumulation was observed with TD feeding regardless of gender. In addition, there was a TD × gender interaction effect (*p* ≤ 0.05) on some carcass zoometric measurements where TD in males decreased carcass length and thorax depth by 4.3 and 4.6%, respectively; conversely, in females, it led to an increase in thorax depth by 3.6% without affecting their carcass length (Figure 6).

The TD × gender interaction did not affect either the non-carcass component percentages (Table 5), wholesale cut yields (Table 6), or meat quality (Table 7). Similarly, supplemental TD alone did not affect (*p* ≥ 0.14) all these variables.

### 3.3. Effects of Gender

Male lambs, compared to ewe lambs, recorded 11.5 bpm less (*p* < 0.01) and had higher (*p* ≤ 0.04) surface temperature on the nostrils (+0.36 °C), ribs (+0.47 °C) and anal region (+0.39 °C), likewise tended (*p* = 0.08) to have higher temperature on the loin, belly, rump and forelimbs (Table 2). There was no effect (*p* ≥ 0.12) of gender on RT and the rest of body surface temperatures. Overall, male lambs had 31, 33, and 25% more (*p* < 0.01) ADG, TWG, and feed efficiency than ewe lambs, respectively (Table 3). This response resulted in male lambs weighing 3.70 kg more (*p* < 0.01) than ewe lambs at the end of the fattening trial. In carcass (Table 4), the gender did not affect (*p* ≥ 0.11) HCW, CCW, *LT* muscle area, and most morphometric measurements (i.e., chest circumference, leg length and circumference). Ewe lambs recorded 6.8% less (*p* = 0.04) EBW but higher (*p* = 0.01) carcass yield (47.66 vs. 49.74%) than male lambs. In addition, higher (*p* < 0.01) backfat thickness, KPH fat, and mesenteric fat were observed in lambs than in male lambs; females tended (*p* = 0.10) also to accumulate more omental fat.

On the other hand, no effect of gender (*p* ≥ 0.14) was observed on the weights (expressed as % of EBW) of blood, skin, lungs, liver, heart, spleen, and empty gastrointestinal tract (Table 5). Male lambs versus ewe lambs presented higher (*p* ≤ 0.04) head (5.19 vs. 6.10%) and kidney (0.29 vs. 0.32%) weights and also tended to have higher (*p* = 0.08) feet weights. In wholesale cuts (Table 6), the shoulder yield was higher (*p* < 0.01) by 2.29%, while the forequarter and neck yields tended to be higher (*p* = 0.10) in male lambs than in ewe lambs. Conversely, the rib (*p* = 0.10), hindquarter (*p* = 0.10), and plain loin (*p* = 0.06) yields tended to be higher in ewe lambs. The rest of the cut yields were similar (*p* ≥ 0.21) between genders. Finally, the pH in male meat was lower (*p* = 0.04) at 45 min (6.36 vs. 6.49) and higher (*p* = 0.01) at 24 h postmortem (5.82 vs. 5.94) compared to that recorded in female meat (Table 7). In meat aged for 7 d, the gender only affected the a* and C* values, with a* being higher (*p* = 0.05) and C* tending to be higher (*p* = 0.09) in male meat than in female meat. The remaining color variables, as well as the WHC, cooking loss, and shear force, did not vary (*p* ≥ 0.11) due to gender.

## 4. Discussion

### 4.1. Environmental Conditions

Sheep of this study were exposed to a natural hot environment (Ta = ~34 °C), characteristic of the summer season in desert regions. Despite the good adaptation by hair sheep to warm environments, these breeds present imminent signs of thermal discomfort at Ta = 32 °C, and this becomes very noticeable at Ta ≥ 35 °C [29,30], since heat losses by latent means increase from 55% at 24 °C to 82 and 96% at Ta of 32 and 36 °C, respectively [30]. These climatic conditions provided an HS environment for lambs as the average Ta exceeded the upper limit (i.e., 30 °C) of the thermoneutral zone for hair breed sheep [1]. Based on the average THI, lambs experienced extremely severe HS (THI > 25.6 vs. 35.9 units) [26]. It is noteworthy that lambs were continuously exposed throughout the day to this type of HS, since THI fluctuated between 29 (early morning) and 43 units (afternoon). Therefore, HS mitigation strategies are needed in intensive sheep meat production of this area.

### 4.2. Effects of Thiamine Diphosphate

TD is a metabolic enzyme cofactor that, in addition to promoting glucose production and energy efficiency, has antioxidant and anti-inflammatory action in mammals [5,6]. To date, this compound has been investigated for improving energy efficiency in sheep raised under thermoneutral conditions, while no reports have been found in hot environments. Healthy sheep maintain a core temperature between 38.3 and 39.9 °C in a thermoneutral environment [26], and HS conditions can raise it due to inefficient thermoregulatory ability [3,31]. Here, the fattening lambs, irrespective of gender, experienced mild hyperthermia [32] as their average RT exceeded the upper limit of the normal range by 0.2 °C (39.9 vs. 40.1 °C) [26], and feeding TD did not prevent it. Despite this response, TD-fed lambs exhibited different physiological thermoregulation strategies, given that they dissipated more body heat load through the skin than control lambs. These findings might be related to the fact that TD improves both the thermogenic capacity of subcutaneous fat tissue adipocytes [33] and peripheral vasodilation [34,35].

Supplemental TD in ewe lambs, but not in male lambs, increased body heat losses through the eyes, forehead, and ears, which indicates a gender-dependent effect of TD on head thermoregulation in heat-stressed hair lambs. Male lambs secrete testosterone, which has a vasodilator function in the brain according to studies with murine models [36]. Thiamine, for its part, plays an essential role in maintaining optimal brain function in ruminants by supplying it with glucose and ensuring adequate blood circulation [35,37]. As a result, this glucogenic cofactor aids in improving blood flow, endothelial function, and vasodilatation in the head [35,38], which explains the positive impact of TD on the heat losses across eyes, ears, and forehead in ewe lambs. In male lambs, this beneficial effect of TD was not observed because testosterone likely activated a thermoregulatory mechanism in the head, like that of TD. Thus, control male lambs achieved similar temperatures in those head regions as TD-fed male and female lambs.

Overall, the results partially support the original hypothesis of this research because the thermoregulatory capacity of the lambs was not compromised by TD supplementation. However, no benefit was observed in growth rate, economically important carcass characteristics, or meat quality with the inclusion of this feed additive during the fattening period. In line with this responses, different studies in sheep housed in a thermoneutral environment did not find changes in growth traits and feed efficiency with oral or injected thiamine [10,13,39]. In fattening calves, the dietary inclusion of this glucogenic cofactor also did not improve productive performance [40,41], carcass characteristics, or meat quality [40]. Therefore, the available evidence shows that the effect of TD on energy metabolism in ruminants is not strong enough to promote growth and affect meat quality. In fact, Kalyesubula et al. [10] mention that TD in sheep could function more as a redistributor of body energy than as a provider of energy substrate for muscle mass formation.

Unexpectedly, TD reduced DMI only in ewe lambs without affecting growth rate, feed efficiency, carcass weight, or *LT* area. Thiamine can increase dietary energy efficiency by functioning as a pyruvate dehydrogenase coenzyme in the glycolysis pathway for the acetyl CoA formation, a substrate that produces ATP energy [6,7]. It is also a cofactor of catabolic enzymes abundant in some organs, muscles, and fatty tissue, which degrade these tissues to release gluconeogenic precursors (i.e., short-chain branched-chain amino acids and non-esterified fatty acids) that promote the uptake of hepatic glycogen and body energy availability [8,39]. Given that TD did not affect fat deposition (except mesenteric fat), organ weights, and wholesale cut yields in the present study, it is speculated that this glucogenic cofactor improved dietary energy efficiency in ewe lambs. Consequently, they met their energy requirements for growth with lower DMI. Similarly, castrated and ewe lambs had a reduction in feed intake when TD was supplemented at doses greater than 100 mg/d/animal [13], while in intact bulls, does not [40].

In contrast to these findings, it had been hypothesized that hair-breed male lambs would have a better productive response to TD supplementation than ewe lambs under the climatic conditions of the present study. There is no clear explanation as to why the opposite happened, considering that the metabolic sexual dimorphism effect was expected to prevail in these hair sheep as their gonads maintain endocrine activity during the hot summer season in arid regions [18,19]. Perhaps the improved dietary energy efficiency observed in TD-fed ewe lambs could be linked to the fact that females were more thermotolerant than males. In this regard, a sexually dimorphic stress response has been reported in Brahman cattle, a bovine breed recognized for its high tolerance to HS, similar to hair sheep [42]. Compared to bulls, Brahman heifers demonstrated greater stress resilience, as they recorded higher circulating cortisol concentrations and pro-inflammatory activity but did not experience immunosuppression. In addition, further research is needed to clarify whether the optimal dose of TD is dependent on gender to exert beneficial effects on dietary energy efficiency in heat-stressed sheep.

### 4.3. Effects of Gender

The high summer Ta recorded in the present study compromised the thermoregulatory capacity in lambs of both genders, leading them to experience mild hyperthermia as their RT were 0.25 °C above the normal range [1]. Gender did not affect RT, suggesting that this genetic factor does not influence the thermotolerance of hair breed lambs fattened in desert climate. Although the physiological thermoregulation strategy used by these sheep was gender dependent. Female lambs dissipated excess body heat load by making more use of evaporative mechanisms (e.g., higher RR) than sensible losses. In contrast, the opposite occurred in male lambs who favored greater heat radiation across the skin in varies body regions. This finding is consistent with results observed in heat stressed sheep from other breeds, where females required a greater increase in RR than males to maintain normothermia [43]. The difference between gender in the activation of physiological thermoregulation mechanisms may be attributed to several things: (1) sexual dimorphisms in physiological variables; (2) females tend to deposit more subcutaneous fat, which reduces their ability to transfer heat through the skin [44]; and (3) testosterone synthesized by males promotes peripheral vasodilation in response to elevated Ta, facilitating the redistribution of blood flow to peripheral tissues to dissipate heat load through the skin [45].

As expected, given some previous antecedents [46,47,48,49], male lambs showed superior growth and feed efficiency despite the high Ta recorded. It is noteworthy that male lambs weighed 3.7 kg more at slaughter than ewe lambs; however, this advantage was not reflected in carcass weight, and, in fact, they had a 2.1% lower carcass yield. These results could be explained by differences in body composition between genders [50]. So, the difference in slaughter weight in favor of male sheep was due to a greater BW associated with the full gastrointestinal tract, head, and some offal (i.e., testicles, feet, and kidney). Overall, carcass weight and yield results showed that HS reduces the natural efficiency of hair-breed male lambs, but not in ewe lambs, for gaining BW associated with better carcass mass deposition. This finding is not surprising, considering that HS can minimize testicular endocrine function in male lambs, leading to reduction in circulating concentrations of testosterone [51]. This steroidal hormone represents a natural anabolic for male sheep, as it stimulates the deposition of lean tissue and improves growth and carcass weight [52].

Furthermore, ewe lambs deposited markedly more internal and dorsal fat than male lambs, which is consistent with other studies with hair breed sheep under thermoneutral conditions [47,48,49,50]. The elevated lipogenic capacity exhibited by ewes is attributable to their ovarian estrogen production [16]. It is worth noting that steroid hormones play an essential role in the development of secondary sexual characteristics in sheep, which is directly reflected in palpable differences in the body conformation of males and females [52]. Testosterone in male lambs promotes longer bodies with a wider rib cage and more muscular chest and neck, while estrogen in ewe lambs produces wider hips with a narrower chest and a thinner neck [16]. This could explain the results of wholesale cut yields where male lambs had greater yields in forequarter, neck, and shoulder; conversely, female lambs tended to have better yields in hindquarter and plain loin.

Finally, it has been documented that sheep meat quality can vary according to gender, as male lambs reach physiological maturity earlier and have greater muscle glycogen reserves [15]. However, research results on sheep in this regard are still inconsistent under thermoneutral conditions [53,54], and no reports were found on hair breed sheep under HS conditions. Here, the gender modified the pH of the *LT* muscle in the first 24 h postmortem; particularly, the male muscle had a higher ultimate pH than the female muscle, which was above the normal range (5.4–5.8) [55]. Based on this muscle pH, one might expect a deterioration in meat quality because high ultimate pH is associated with the presence of dark, firm, and dry meat [56]. But the male meat only had a slightly redder tone with no difference in lightness, shear force, WHC, and cooking loss compared to the female meat. In fact, the average values of these quality variables were within the reference range for sheep meat (WBSF < 49 N, L* = 34 to 44, a* > 14, cooking loss = 15 and 24% and WHC ≥ 80%) [55]. Perhaps the male meat quality did not decrease because the evaluation was made on aged meat. Aging sheep meat for 7 d is a commonly used method to simulate shelf life, but it also has a positive impact by improving meat quality attributes in terms of pH, tenderness, WHC, and sensory profile [57]. This response suggests that future experiments could be conducted using unaged meat to elucidate the pure effect of gender on the meat quality of hair breed sheep subjected to HS.

## 5. Conclusions

Overall, regardless of gender, thiamine diphosphate supplementation did not prevent hyperthermia or improve growth rate, carcass characteristics, or meat quality of hair breed lambs finished in feedlots under heat stress conditions in desert regions. However, this glucogenic cofactor was effective in decreasing feed intake in ewe lambs, but not in male lambs, without compromising weight gain, carcass weight, *Longissimus thoracis* muscle area, and meat quality. Furthermore, thiamine diphosphate enhanced sensible heat losses in male and female lambs. Therefore, thiamine diphosphate supplementation proved to be a suitable heat stress mitigation strategy in hair ewe lambs, as it increased dietary energy efficiency.

## Figures and Tables

**Table 1:** Ingredients and chemical analysis of the basal experimental diet offered to lambs.

Ingredients ^1^	% Mixed Basis	Chemical Composition ^2^	% DM Basis
Alfalfa hay	12.5	Dry matter	90.5
Wheat straw	15.0	Crude protein	16.2
Ground wheat grain	60.0	Ether extract	1.50
Soybean meal	10.0	Crude fiber	8.30
Mineral-vitamin premix	2.00	Acid detergent fiber	18.0
Calcium bicarbonate	0.50	Neutral detergent fiber	25.7
		Ash	7.80
		Calculates net energy	Mcal/kg DM
		Maintenance	1.83
		Gain ^3^	1.06

^1^ Vitamin-mineral premix contained: vitamin A = 1,200,000 IU; vitamin D3 = 200,000 IU; vitamin D-3 = 1200 IU; vitamin E = 3.2 mg; riboflavin = 1 g; vitamin K-3 = 0.4 g; niacin = 4 g; calcium pantothenate = 2 g; choline chloride = 30 g; Zinc = 16 g; Iron = 16 g; Copper = 1 g; Manganese = 1 g; Iodine = 90 mg; Selenium = 33 mg; butylhydroxytoluene = 3.3 g. ^2^ Except for dry matter (DM) percentage, all compounds were determined as a DM percentage. ^3^ Calculated based on energy values specified by the NRC [25] for each ingredient.

**Table 2:** Effect of thiamine diphosphate (TD) and gender (G) on physiological variables of Dorper × Katahdin lambs fattened under heat stress conditions.

Items ^2^	Thiamine Diphosphate ^1^			Gender			*p*-Values					
	Without	With	SEM	Male	Females	SEM	TD	G	H	TD × G	TD × H	G × H
RT (°C)	40.2	40.1	0.09	40.1	40.2	0.09	0.79	0.83	<0.01	0.37	0.49	0.75
RR (bpm)	142	144	2.27	137 a	149 b	1.27	0.58	<0.01	<0.01	0.33	0.67	0.27
Body surface temperatures (°C)												
Eye	38.5	38.8	0.12	38.8	38.5	0.13	0.09	0.18	<0.01	0.04	0.52	0.11
Ear	37.9	38.1	0.09	38.0	37.7	0.10	0.07	0.91	<0.01	0.05	0.86	0.12
Muzzle	36.7	36.7	0.12	36.8	36.7	0.12	0.61	0.66	<0.01	0.49	0.31	0.49
Forehead	38.0 a	38.4 b	0.10	38.3 b	38.0 a	0.10	0.04	0.04	<0.01	0.05	0.34	0.20
Nostrils	37.9	38.1	0.11	38.2 b	37.8 a	0.11	0.18	0.04	<0.01	0.32	0.18	0.27
Neck	37.6 a	38.0 b	0.12	37.9	37.7	0.12	0.04	0.17	<0.01	0.54	0.54	0.11
Shoulder	37.9 a	38.4 b	0.15	38.3	38.0	0.15	0.05	0.19	<0.01	0.52	0.44	0.12
Loin	37.6 a	38.0 b	0.13	38.0	37.7	0.13	0.05	0.08	<0.01	0.17	0.65	0.12
Ribs	37.9	38.2	0.15	38.3 b	37.8 a	0.15	0.09	0.04	<0.01	0.24	0.65	0.11
Belly	37.9	38.3	0.17	38.3	37.8	0.17	0.12	0.08	<0.01	0.46	0.33	0.11
Flank	37.3	37.7	0.15	37.6	37.4	0.15	0.07	0.20	<0.01	0.22	0.35	0.11
Rump	37.5 a	37.9 b	0.12	37.8	37.6	0.12	0.02	0.08	<0.01	0.27	0.65	0.12
Leg	37.3	37.8	0.20	37.8	37.3	0.20	0.11	0.12	<0.01	0.64	0.33	0.14
Fore limb	37.2 a	37.7 b	0.11	37.6	37.3	0.11	0.01	0.08	<0.01	0.12	0.39	0.20
Hind limb	37.3	37.6	0.13	37.6	37.3	0.13	0.09	0.12	<0.01	0.60	0.33	0.28
Anal	39.2 a	39.7 b	0.11	39.6 b	39.2 a	0.11	<0.01	0.02	<0.01	0.37	0.56	0.67
Vulva	38.2 a	38.9 b	0.09	---	---	---	<0.01	---	<0.01	---	0.18	---
Perineum	37.9 a	38.7 b	0.13	---	---	---	<0.01	---	<0.01	---	0.59	---
Testicles	33.8 a	35.4 b	0.25	---	---	---	0.01	---	<0.01	---	0.34	---

Within each factor, different letters (a, b) indicate significant differences at *p* ≤ 0.05. ^1^ Lambs fed a basal diet with 0 (without) or 250 (with) mg of TD/kg feed. ^2^ Table abbreviations: RT = Rectal temperature, RR = Respiratory rate (respirations per minute), SEM = Standard error of the means; H = Hour of the day.

**Table 3:** Effect of thiamine diphosphate (TD) and gender (G) on feedlot performance of Dorper × Katahdin lambs fattened under heat stress conditions.

Items ^2^	Thiamine Diphosphate ^1^			Gender			*p*-Values		
	Without	With	SEM	Males	Females	SEM	TD	G	TD × G
Initial weight (kg)	31.5	31.5	0.40	31.6	31.4	0.40	0.94	0.70	0.92
Final weight (kg)	40.2	40.5	0.67	42.2 b	38.5 a	0.67	0.70	<0.01	0.58
Daily weight gain (g/d)	223	235	12.4	261 b	183 a	12.6	0.58	<0.01	0.43
Total weight gain (kg)	8.76	8.99	0.41	10.6 b	7.09 a	0.41	0.58	<0.01	0.43
Dry matter intake (kg/d)	1.28	1.24	0.03	1.33 b	1.19 a	0.03	0.28	<0.01	0.02
Feed efficiency (g/kg)	174	189	7	197 b	153 a	11.5	0.30	<0.01	0.65

Within each factor, different letters (a, b) indicate differences at *p* ≤ 0.05. ^1^ Lambs fed a basal diet with 0 (without) or 250 (with) mg of TD/kg feed. ^2^ Table abbreviations: SEM = Standard error of the means.

**Table 4:** Effect of thiamine diphosphate (TD) and gender (G) on carcass characteristics of Dorper × Katahdin lambs fattened under heat stress conditions.

Items ^2^	Thiamine Diphosphate ^1^			Gender			*p*-Values		
	Without	With	SEM	Males	Females	SEM	TD	G	TD × G
Empty live weight (kg)	33.7	34.1	0.71	35.1 b	32.7 a	0.71	0.71	0.04	0.70
Hot carcass weight (kg)	18.9	19.1	0.44	19.4	18.6	0.44	0.80	0.24	0.83
Cold carcass weight (kg)	17.9	18.5	0.47	18.7	17.6	0.48	0.42	0.15	0.77
Dressing (%)	48.9	48.5	0.38	47.7 a	49.7 b	0.38	0.57	<0.01	0.53
*LT* area (cm^2^)	17.9	18.2	1.41	18.6	17.4	1.43	0.86	0.57	0.36
Carcass length (cm)	63.3	62.3	0.63	64.2 b	61.3 a	0.64	0.25	0.01	0.05
Thorax depth (cm)	16.8	16.7	0.17	16.9	16.7	0.17	0.73	0.47	0.02
Chest circumference (cm)	67.6	68.3	0.67	68.8	67.1	0.68	0.44	0.11	0.15
Leg length (cm)	32.2	31.4	0.78	32.5	31.0	0.79	0.49	0.24	0.71
Leg circumference (cm)	42.8	43.0	0.73	42.2	43.5	0.74	0.91	0.26	0.69
**Body fat**									
Backfat thickness (mm)	3.00	2.82	0.40	1.80 a	4.02 b	0.40	0.76	<0.01	0.69
KPH (%)	2.25	2.27	0.11	1.96 a	2.56 b	0.11	0.90	<0.01	0.74
Omental (%)	2.98	3.19	0.17	2.87	3.30	0.17	0.39	0.10	0.50
Mesenteric (%)	2.30 b	1.80 a	0.14	1.69 a	2.41 b	0.14	0.03	<0.01	0.67

Within each factor, different letters (a, b) indicate differences at *p* ≤ 0.05. ^1^ Lambs fed a basal diet with 0 (without) or 250 (with) mg of TD/kg feed. ^2^ Table abbreviations: *LT* = *Longissimus thoracic*, KPH = Kidney-pelvic-heart fat, SEM = Standard error of the means.

**Table 5:** Effect of thiamine diphosphate (TD) and gender (G) on non-carcass components of Dorper × Katahdin lambs fattened under heat stress conditions.

Items ^2^	Thiamine Diphosphate ^1^			Gender			*p*-Values		
	Without	With	SEM	Males	Females	SEM	TD	G	TD × G
Head (%)	5.55	5.74	0.17	6.10 b	5.19 a	0.17	0.44	<0.01	0.51
Blood (%)	4.45	4.39	0.12	4.55	4.30	0.12	0.72	0.17	0.56
Feet (%)	2.60	2.52	0.04	2.61	2.51	0.04	0.14	0.08	0.19
Skin (%)	11.3	11.4	0.31	11.2	11.5	0.31	0.90	0.52	0.90
Kidney (%)	0.31	0.30	0.01	0.32 b	0.29 a	0.01	0.35	0.04	0.75
Lung (%)	1.51	1.42	0.07	1.50	1.44	0.07	0.18	0.77	0.82
Liver (%)	2.16	2.12	0.05	2.20	2.09	0.05	0.50	0.14	0.47
Heart (%)	0.40	0.40	0.01	0.39	0.41	0.01	0.76	0.36	0.85
Spleen (%)	0.23	0.23	0.04	0.23	0.23	0.04	0.95	0.98	0.38
Empty GT (%)	5.46	5.41	0.14	5.64	5.22	0.14	0.33	0.22	0.62
Testicles (%)	1.73	1.85	0.04	---	---	---	0.23	---	---

Within each factor, different letters (a, b) indicate differences at *p* ≤ 0.05. ^1^ Lambs fed a basal diet with 0 (without) or 250 (with) mg of TD/kg feed. ^2^ Weights expressed as a percentage of the empty body weight. Table abbreviations: GT = Empty gastrointestinal tract, SEM = Standard error of the means.

**Table 6:** Effect of thiamine diphosphate (TD) and gender (G) on wholesale cut yields of Dorper × Katahdin lambs fattened under heat stress conditions.

Items ^2^	Thiamine Diphosphate ^1^			Gender			*p*-Values		
	Without	With	SEM	Males	Females	SEM	TD	G	TD × G
Forequarter (%)	52.9	52.6	0.62	53.6	51.9	0.63	0.81	0.10	0.84
Neck (%)	3.68	3.49	0.13	3.77	3.41	0.14	0.35	0.10	0.25
Shoulder (%)	31.0	31.6	0.41	32.5 b	30.2 a	0.42	0.35	<0.01	0.88
Loin (%)	8.11	7.43	0.43	7.76	7.77	0.44	0.29	0.99	0.81
Ribs (%)	10.0	10.1	0.38	9.54	10.6	0.39	0.92	0.10	0.98
Hindquarter (%)	47.1	47.3	0.62	46.4	48.1	0.63	0.81	0.10	0.84
Leg (%)	33.2	33.5	0.47	33.3	33.5	0.48	0.67	0.82	0.77
Plain loin (%)	8.96	8.90	0.40	8.33	9.53	0.40	0.92	0.06	0.99
Breast and flank (%)	4.96	4.93	0.18	4.80	5.08	0.18	0.69	0.21	0.36

Within each factor, different letters (a, b) indicate differences at *p* ≤ 0.05. ^1^ Lambs fed a basal diet with 0 (without) or 250 (with) mg of TD/kg feed. ^2^ Weights expressed as a percentage of the half carcass. Table abbreviations: SEM = Standard error of the means.

**Table 7:** Effect of thiamine diphosphate (TD) and gender (G) on the aged meat quality of Dorper × Katahdin lambs fattened under heat stress conditions.

Items ^2^	Thiamine Diphosphate ^1^			Gender			*p*-Values		
	Without	With	SEM	Males	Females	SEM	TD	G	TD × G
pH postmortem									
45 min	6.42	6.43	0.04	6.36 a	6.49 b	0.04	0.85	0.04	0.40
24 h	5.89	5.87	0.02	5.94 b	5.82 a	0.02	0.70	0.01	0.44
Meat aged for 7 d									
pH	5.62	5.53	0.04	5.62	5.54	0.04	0.16	0.23	0.82
Redness (a*)	21.9	22.0	0.25	22.4 b	21.6 a	0.25	0.80	0.05	0.11
Yellowness (b*)	7.02	7.46	0.46	7.47	7.01	0.46	0.50	0.51	0.41
Lightness (L*)	41.2	41.7	0.73	42.3	40.7	0.74	0.66	0.17	0.51
Hue angle (H*)	17.8	18.8	0.85	18.6	18.0	0.87	0.45	0.64	0.54
Chroma (C*)	23.1	23.3	0.35	23.7	22.7	0.35	0.61	0.09	0.15
WHC (%)	90.3	89.7	0.73	90.9	89.2	0.74	0.54	0.14	0.83
WBSF (N)	37.6	40.2	1.51	38.9	39.0	1.51	0.26	0.95	0.27
Cooking loss (%)	22.1	22.2	2.02	21.2	23.1	2.05	0.99	0.53	0.28

Within each factor, different letters (a, b) indicate differences at *p* ≤ 0.05. ^1^ Lambs fed a basal diet with 0 (without) or 250 (with) mg of TD/kg feed. ^2^ Table abbreviations: WHC = Water-holding capacity, WBSF = Warner-Bratzler shear force, SEM = Standard error of the means.

## Data Availability

The datasets used in this research are available from the corresponding author upon reasonable request.

## References

[1] Vicente Pérez R., Macías Cruz U., Avendaño Reyes L., Correa Calderón A., López Baca M.D.l.Á., Lara Rivera A.L. (2020). Impacto Del Estrés Por Calor En La Producción de Ovinos de Pelo. Revisión. Rev. Mex. Cienc. Pecu..

[2] Macías-Cruz U., Avendaño-Reyes L., Álvarez-Valenzuela F.D., Torrentera-Olivera N.G., Meza-Herrera C., Mellado-Bosque M., Correa-Calderón A. (2013). Growth and Carcass Characteristics of Ewe Lambs Treated with Zilpaterol Hydrochloride during Spring and Summer. Rev. Mex. Cienc. Pecu..

[3] Macías-Cruz U., Saavedra O.R., Correa-Calderón A., Mellado M., Torrentera N.G., Chay-Canul A., López-Baca M.A., Avendaño-Reyes L. (2020). Feedlot Growth, Carcass Characteristics and Meat Quality of Hair Breed Male Lambs Exposed to Seasonal Heat Stress (Winter vs. Summer) in an Arid Climate. Meat Sci..

[4] Nicolás-López P., Macías-Cruz U., Mellado M., Correa-Calderón A., Meza-Herrera C.A., Avendaño-Reyes L. (2021). Growth Performance and Changes in Physiological, Metabolic and Hematological Parameters Due to Outdoor Heat Stress in Hair Breed Male Lambs Finished in Feedlot. Int. J. Biometeorol..

[5] Serra M., Mollace R., Ritorto G., Ussia S., Altomare C., Tavernese A., Preianò M., Palma E., Muscoli C., Mollace V. (2025). A Systematic Review of Thiamine Supplementation in Improving Diabetes and Its Related Cardiovascular Dysfunction. Int. J. Mol. Sci..

[6] Manzetti S., Zhang J., van der Spoel D. (2014). Thiamin Function, Metabolism, Uptake, and Transport. Biochemistry.

[7] Hrubša M., Siatka T., Nejmanová I., Vopršalová M., Krčmová L.K., Matoušová K., Javorská L., Macáková K., Mercolini L., Remião F. (2022). Biological Properties of Vitamins of the B-Complex, Part 1: Vitamins B1, B2, B3, and B5. Nutrients.

[8] Mann G., Mora S., Madu G., Adegoke O.A.J. (2021). Branched-Chain Amino Acids: Catabolism in Skeletal Muscle and Implications for Muscle and Whole-Body Metabolism. Front. Physiol..

[9] Kaźmierczak-Barańska J., Halczuk K., Karwowski B.T. (2025). Thiamine (Vitamin B1)—An Essential Health Regulator. Nutrients.

[10] Kalyesubula M., Mopuri R., Asiku J., Rosov A., Yosefi S., Edery N., Bocobza S., Moallem U., Dvir H. (2021). High-Dose Vitamin B1 Therapy Prevents the Development of Experimental Fatty Liver Driven by Overnutrition. DMM Dis. Models Mech..

[11] Ma Y., Yang P., Li P., Elsabagh M., Cheng L., Chen H., Feng Y., Li Z., Xu M. (2024). Dietary Thiamine Supplementation Modulates Ruminal Microbiota and Partly Restores Lactation Performance in Lactating Hu Ewes under Heat-Stress Conditions. Anim. Feed. Sci. Technol..

[12] McManus C.M., Lucci C.M., Maranhão A.Q., Pimentel D., Pimentel F., Rezende Paiva S. (2022). Response to Heat Stress for Small Ruminants: Physiological and Genetic Aspects. Livest. Sci..

[13] Neville B.W., Schauer C.S., Karges K., Gibson M.L., Thompson M.M., Kirschten L.A., Dyer N.W., Berg P.T., Lardy G.P. (2010). Effect of Thiamine Concentration on Animal Health, Feedlot Performance, Carcass Characteristics, and Ruminal Hydrogen Sulfide Concentrations in Lambs Fed Diets Based on 60% Distillers Dried Grains plus Solubles. J. Anim. Sci..

[14] Clarke S.D., Clarke I.J., Rao A., Cowley M.A., Henry B.A. (2012). Sex Differences in the Metabolic Effects of Testosterone in Sheep. Endocrinology.

[15] Gallo C., Tarumán J., Larrondo C. (2018). Main Factors Affecting Animal Welfare and Meat Quality in Lambs for Slaughter in Chile. Animals.

[16] Schumacher M., Delcurto-Wyffels H., Thomson J., Boles J. (2022). Fat Deposition and Fat Effects on Meat Quality—A Review. Animals.

[17] van Wettere W.H.E.J., Kind K.L., Gatford K.L., Swinbourne A.M., Leu S.T., Hayman P.T., Kelly J.M., Weaver A.C., Kleemann D.O., Walker S.K. (2021). Review of the Impact of Heat Stress on Reproductive Performance of Sheep. J. Anim. Sci. Biotechnol..

[18] Macías-Cruz U., Gastélum M.A., Álvarez F.D., Correa A., Díaz R., Meza-Herrera C.A., Mellado M., Avendaño-Reyes L. (2016). Effects of Summer Heat Stress on Physiological Variables, Ovulation and Progesterone Secretion in Pelibuey Ewes under Natural Outdoor Conditions in an Arid Region. Anim. Sci. J..

[19] Barragán A.L., Avendaño-Reyes L., Mellado-Bosque M., Meza-Herrera C.A., Vicente-Pérez R., Castañeda V.J., Díaz-Molina R., Macías-Cruz U. (2023). Seasonal Heat Stress Compromises Testicular Thermoregulation and Semen Quality of Dorper Rams Raised in a Desert Climate. J. Therm. Biol..

[20] Theusme C., Avendaño-Reyes L., Macías-Cruz U., Correa-Calderón A., García-Cueto R.O., Mellado M., Vargas-Villamil L., Vicente-Pérez A. (2021). Climate Change Vulnerability of Confined Livestock Systems Predicted Using Bioclimatic Indexes in an Arid Region of México. Sci. Total Environ..

[21] NOM-051-ZOO-1995. https://www.gob.mx/senasica/documentos/nom-051-zoo-1995.

[22] NOM-062-ZOO-1999. https://www.gob.mx/cms/uploads/attachment/file/203498/NOM-062-ZOO-1999_220801.pdf.

[23] NOM-033-SAG/ZOO-2014. https://www.gob.mx/cms/uploads/attachment/file/133499/4.-_NORMA_OFICIAL_MEXICANA_NOM-033-SAG-ZOO-2014.pdf.

[24] NRC (2007). Nutrient Requirements of Small Ruminants.

[25] NRC (1985). Nutrient Requirements of Sheep.

[26] Marai I.F.M., El-Darawany A.A., Fadiel A., Abdel-Hafez M.A.M. (2007). Physiological Traits as Affected by Heat Stress in Sheep-A Review. Small Rumin. Res..

[27] Avendaño-Reyes L., Macías-Cruz U., Álvarez-Valenzuela F.D., Águila-Tepato E., Torrentera-Olivera N.G., Soto-Navarro S.A. (2011). Effects of Zilpaterol Hydrochloride on Growth Performance, Carcass Characteristics, and Wholesale Cut Yield of Hair-Breed Ewe Lambs Consuming Feedlot Diets under Moderate Environmental Conditions. J. Anim. Sci..

[28] Sutton D.S., Ellis M., Lan Y., McKeith F.K., Wilson E.R. (1997). Influence of Slaughter Weight and Stress Gene Genotype on the Water-Holding Capacity and Protein Gel Characteristics of Three Porcine Muscles. Meat Sci..

[29] Rodrigues R.C.M., Araújo Furtado D., Ribeiro N.L., Silva R.d.S., de Oliveira A.G., da Costa Silva J.A.P., da Silva M.R., Mascarenhas N.M.H., Cavalcanti C.R., Junqueira Ayres G.D. (2024). Thermoneutral Temperature and Thermal Stress of Sheep Creole: Physiological and Ingestive Behaviour. J. Appl. Anim. Res..

[30] Silva R.d.S., Furtado D.A., Ribeiro N.L., Neto J.P.L., Rodrigues R.C.M., Oliveira A.G.d., Silva J.A.P.d.C., Silva M.R.d., Mascarenhas N.M.H., Marques J.I. (2024). Physiological Variables and Estimates of Heat Exchange in Sheep Kept at Thermoneutral and Thermal Stress Temperatures. Small Rumin. Res..

[31] Macías-Cruz U., López-Baca M.A., Vicente R., Mejía A., Álvarez F.D., Correa-Calderón A., Meza-Herrera C.A., Mellado M., Guerra-Liera J.E., Avendaño-Reyes L. (2016). Effects of Seasonal Ambient Heat Stress (Spring vs. Summer) on Physiological and Metabolic Variables in Hair Sheep Located in an Arid Region. Int. J. Biometeorol..

[32] Sejian V., Krishnan G., Bagath M., Vaswani S., Pragna P., Aleena J., Lees A.M., Maurya V.P., Bhatta R. (2017). Measurement of Severity of Heat Stress in Sheep. Sheep Production Adapting to Climate Change.

[33] Vinnai B.Á., Arianti R., Fischer-Posovszky P., Wabitsch M., Fésüs L., Kristóf E. (2025). The Importance of Thiamine Availability in the Thermogenic Competency of Human Adipocytes. Mol. Cell. Endocrinol..

[34] Yasmin F., Ali S.H., Naeem A., Savul S., Afridi M.S.I., Kamran N., Fazal F., Khawer S., Savul I.S., Najeeb H. (2022). Current Evidence and Future Perspectives of the Best Supplements for Cardioprotection: Have We Reached the Final Chapter for Vitamins?. Rev. Cardiovasc. Med..

[35] Goma A.A. (2025). Thiamine Deficiency and Brain Degeneration in Calves. Anim. Sci. Cases.

[36] Shvareva N., Kaplanski J., Abramovich L., Sod-Moriah U.A. (1998). Testosterone Modifies Response to Chronic Heat Exposure in Rats. Comp. Biochem. Physiol. A Mol. Integr. Physiol..

[37] Alsaad K.M., Dhahi J.H., Tawfiq S.I. (2020). Polioencephalomalacia Caused by Thiamine Deficiency in Sheep of Basrah Province, Iraq. Egypt. J. Vet. Sci..

[38] Hakim A.M. (1984). The Induction and Reversibility of Cerebral Acidosis in Thiamine Deficiency. Ann. Neurol..

[39] Kalyesubula M., Mopuri R., Rosov A., Van Bommel G., Dvir H. (2021). Metabolic Effects of Vitamin B1 Therapy under Overnutrition and Undernutrition Conditions in Sheep. Nutrients.

[40] Rosas Aragón J. (2018). Respuesta Productiva, Costo de Producción, Calidad de la Canal y de la Carne de Toretes Suplementados con Difosfato de Tiamina y β-Agonistas. Master’s Thesis.

[41] Silzell S.A., Hellwig D.H., Kegley E.B., Coffey K.P., Beers K., Daniels L.B. (2002). Effects of Supplemental Thiamin on Growth Performance and Immune Function in Stressed Stocker Cattle1. J. Appl. Anim. Res..

[42] Hulbert L.E., Carroll J.A., Ballou M.A., Burdick N.C., Dailey J.W., Caldwell L.C., Loyd A.N., Vann R.C., Welsh T.H., Randel R.D. (2013). Sexually Dimorphic Stress and Pro-Inflammatory Cytokine Responses to an Intravenous Corticotropin-Releasing Hormone Challenge of Brahman Cattle Following Transportation. Innate Immun..

[43] Iddriss A.R.I., Rahim A.A. (2018). Heat Tolerance in Djallonke Sheep under Guinea Savannah Conditions. Trop. Agric..

[44] Abbaya H.Y., Philimon Y., Elihu A., Lawal A.U., Lumboyi I.A. (2022). Species, Age and Sex Effect on Thermoregulatory Parameters of Animals in Hot Season of Mubi. J. Biol. Genet. Res..

[45] Fernández-Peña C., Reimúndez A., Viana F., Arce V.M., Señarís R. (2023). Sex Differences in Thermoregulation in Mammals: Implications for Energy Homeostasis. Front. Endocrinol..

[46] Macías-Cruz U., Álvarez-Valenzuela F.D., Rodríguez-García J., Correa-Calderón A., Torrentera-Olivera N.G., Molina Ramírez L., Avendaño-Reyes L. (2010). Growth and Carcass Traits in Pure Pelibuey Lambs and Crosses F1 with Dorper and Katahdin Breeds in Confinement. Arch. Med. Vet..

[47] Tadeu M., Cardoso M., Vieira Landim A., Louvandini H., Mcmanus C. (2013). Performance and Carcass Quality in Three Genetic Groups of Sheep in Brazil. Rev. Bras. Zootec..

[48] Muñoz-Osorio G.A., Aguilar-Caballero A.J., Sarmiento-Franco L.A., Wurzinger M., Sandoval-Castro C.A. (2020). Effect of Two Housing Systems and Sex on Productive Performance of Lamb during the Fattening. Arch. Zootec..

[49] Landim A.V., Roriz N.D., Silveira R.M.F., Vega W.H.O., Costa H.H.A., de Sousa L.C.O., Alves G.C., Ferreira J., Mourão G.B. (2021). Sheep Meat Production in the Brazilian Semi-Arid Region: Crossing between Indigenous Breeds. Trop. Anim. Health Prod..

[50] de Vargas Junior F.M., Martins C.F., dos Santos Pinto G., Ferreira M.B., de Almeida Ricardo H., Leão A.G., Fernandes A.R.M., Teixeira A. (2014). The Effect of Sex and Genotype on Growth Performance, Feed Efficiency, and Carcass Traits of Local Sheep Group Pantaneiro and Texel or Santa Inês Crossbred Finished on Feedlot. Trop. Anim. Health Prod..

[51] Barragán Sierra A., Avendaño-Reyes L., Rivera J.A.H., Vicente-Pérez R., Correa-Calderón A., Mellado M., Meza-Herrera C.A., MacÍas-Cruz U. (2021). Thermoregulation and Reproductive Responses of Rams under Heat Stress. Review. Rev. Mex. Cienc. Pecu..

[52] Lefaucheur L.A. (2010). A Second Look into Fibre Typing—Relation to Meat Quality. Meat Sci..

[53] De Lima Jùnior D., de Carvalho F., Da Silva F., do N Rangel A., Novaes L., Difante G. (2016). Intrinsic Factors Affecting Sheep Meat Quality: A Review. Rev. Colomb. Cien Pecu..

[54] Lee J.H., Wildeus S., Lemma B.B., Kouakou B. (2024). Carcass and Meat Quality Characteristics of Purebred (Hair) and Crossbred (Wool × Hair) Sheep Lambs Grazing Fescue Pasture as Influenced by Breed Type, Sex, and Supplementation. J. Appl. Anim. Res..

[55] Corazzin M., Del Bianco S., Bovolenta S., Piasentier E. (2019). Carcass Characteristics and Meat Quality of Sheep and Goat. More than Beef, Pork and Chicken—The Production, Processing, and Quality Traits of Other Sources of Meat for Human Diet.

[56] Ponnampalam E.N., Hopkins D.L., Bruce H., Li D., Baldi G., Bekhit A.E. (2017). Causes and Contributing Factors to “Dark Cutting” Meat: Current Trends and Future Directions: A Review. Compr. Rev. Food Sci. Food Saf..

[57] Maggiolino A., Forte L., Landi V., Pateiro M., Lorenzo J.M., De Palo P. (2024). Enhancement of Culled Ewes’ Meat Quality: Effects of Aging Method and Time. Food Chem. X.

